# Retraction: Small-Molecule Quinolinol Inhibitor Identified Provides Protection against BoNT/A in Mice

**DOI:** 10.1371/annotation/049f74d0-919f-483c-a488-4740ec0a5f4d

**Published:** 2013-09-05

**Authors:** Padma Singh, Manglesh Kumar Singh, Dilip Chaudhary, Vinita Chauhan, Pranay Bharadwaj, Apurva Pandey, Nisha Upadhyay, Ram Kumar Dhaked

It has been brought to the attention of the PLOS ONE editors that substantial parts of the text in this article were appropriated from text in the following publications:

Identification and biochemical characterization of small-molecule inhibitors of Clostridium botulinum neurotoxin serotype A.

Roxas-Duncan V, Enyedy I, Montgomery VA, Eccard VS, Carrington MA, Lai H, Gul N, Yang DC, Smith LA.

Antimicrob Agents Chemother. 2009 Aug;53(8):3478-86

Eubanks LM, Hixon MS, Jin W, Hong S, Clancy CM, et al. (2007) An in vitro and in vivo disconnect uncovered through high-throughput identification of botulinum neurotoxin A antagonists. Proc Natl Acad Sci USA104: 2602–2607. 

PLOS ONE therefore retracts this article due to the identified case of plagiarism. PLOS ONE apologizes to the authors of the publications above and to the readers.

